# Cognition- and circuit-based dysfunction in a mouse model of 22q11.2 microdeletion syndrome: effects of stress

**DOI:** 10.1038/s41398-020-0687-z

**Published:** 2020-01-28

**Authors:** Anushree Tripathi, Michael Spedding, Esther Schenker, Michael Didriksen, Arnaud Cressant, Therese M. Jay

**Affiliations:** 1 Institute of Psychiatry and Neurosciences of Paris (IPNP), INSERM U1266, Pathophysiology of Psychiatric Disorders, Université de Paris, F-75014 Paris, France; 2 Institut de Recherches Servier, Croissy, France; 3 Spedding Research Solutions SAS, 6 rue Ampere, 78110 Le Vesinet, France, 78110 Le Vesinet, France; 4 H. Lundbeck A/S, Synaptic Transmission, Neuroscience Research DK, Ottiliavej 9, 2500 Valby, Denmark; 5 France Brain@vior SAS, 13 rue des moulins neufs, 28300 Saint-Prest, France; 6 Present Address: Department of Integrative Medical Biology, Umeå University, 90187 Umeå, Sweden; 7 Present Address: UMR 1253, iBrain, Université de Tours, Inserm, Tours, France

**Keywords:** Predictive markers, Learning and memory, Schizophrenia

## Abstract

Genetic microdeletion at the 22q11 locus is associated with very high risk for schizophrenia. The 22q11.2 microdeletion (Df(h22q11)/+) mouse model shows cognitive deficits observed in this disorder, some of which can be linked to dysfunction of the prefrontal cortex (PFC). We used behavioral (*n* = 10 per genotype), electrophysiological (*n* = 7 per genotype per group), and neuroanatomical (*n* = 5 per genotype) techniques to investigate schizophrenia-related pathology of Df(h22q11)/+ mice, which showed a significant decrease in the total number of parvalbumin positive interneurons in the medial PFC. The Df(h22q11)/+ mice when tested on PFC-dependent behavioral tasks, including gambling tasks, perform significantly worse than control animals while exhibiting normal behavior on hippocampus-dependent tasks. They also show a significant decrease in hippocampus-medial Prefrontal cortex (H-PFC) synaptic plasticity (long-term potentiation, LTP). Acute platform stress almost abolished H-PFC LTP in both wild-type and Df(h22q11)/+ mice. H-PFC LTP was restored to prestress levels by clozapine (3 mg/kg i.p.) in stressed Df(h22q11)/+ mice, but the restoration of stress-induced LTP, while significant, was similar between wild-type and Df(h22q11)/+ mice. A medial PFC dysfunction may underlie the negative and cognitive symptoms in human 22q11 deletion carriers, and these results are relevant to the current debate on the utility of clozapine in such subjects.

## Introduction

The de novo copy number variant (CNV) of humanchromosome 22q11.2 is one of the strongest genetic risk factor for development of sporadic schizophrenia^1^. This CNV results in a deletion of 1.5–3 Mbp including ~35–60 known genes^2^, most of which are expressed in the brain. The synteny of the human chromosome 22 to mouse chromosome 16 with a high degree of conservation enables the generation of a mouse model with construct validity. To this end, animal models based on either silencing of single genes or deletion of part of this locus have been validated^3^ with behavioral and cognitive deficits, including spatial and working memory deficits^4,5^, impairment in reversal learning^6^, and fear conditioning^7^. Didriksen et al. recently described a novel mouse model (Df(h22q11)/+) with hemizygous deletion of mouse chromosome 16, that corresponds to the region of human 22q11.2 microdeletion^8^. This model was extensively analysed in a battery of cognitive tasks by partner research groups within the NEWMEDS consortium (Innovative Medicines Initiative Grant agreement number 115008). However, the model shows little of the cognitive impairments associated with neuropsychiatric disorders and task performance was close to that of wild-type (Wt) littermates^9^.

We received the Df(h22q11)/+ mice as a part of the IMI NEWMEDS collaboration and focussed on the functional interaction between the hippocampus and the prefrontal cortex (PFC) that has been reported to be abnormal in animal models for psychiatric risk factors^10–13^ and in schizophrenia patients^14–17^. Studies of different 22q11 animal models have established deficits in synaptic plasticity^18^, long range synchrony^10^ as well as inhibitory transmission^19^ within this circuit. We have previously demonstrated a direct but graded monosynaptic projection from the ventral CA1 to the Prelimbic region (Prl) of the medial PFC (mPFC) in mice^20^. The hippocampal-to-prefrontal cortex (H-PFC) pathway is crucial for tasks involving the functional coordination and contribution of both these regions especially in case of mnemonic, emotional, and cognitive processing as well as goal directed behavior: neuronal plasticity in this pathway is exquisitely sensitive to stress in mice and rats^20,21^ and these effects are reversed by low doses of clozapine in rats, but not all antipsychotics. We previously observed that a low dose of clozapine was optimal for modifying frontal cortex theta rhythms^22,23^ which are considered important for long-range connectivity between the hippocampus and mPFC^24,25^ and a poststress treatment of clozapine (but not haloperidol) at such dose protected H-PFC pathway plasticity from stress-induced disruption^26^.

We therefore hypothesized that the Df(h22q11)/+ mice could replicate the regional specific disturbance of the H-PFC functional connectivity and evaluated this dysfunction in the Df(h22q11)/+ mice. We first tested these animals on behavioral paradigms involving predominantly the PFC, the hippocampus, and the H-PFC interaction. We examined neural plasticity in the H-PFC pathway, and the potential protective effects of the atypical antipsychotic clozapine on stress-induced disruption of the H-PFC LTP (long-term potentiation). As the H-PFC pathway regulates PFC activity and function by modulating interneuron-mediated inhibition of pyramidal neurons in the Prl^27^ we also explored PFC cell subpopulations in Df(h22q11)/+ mice relative to Wt mice.

## Methods

### Animals

All experimental procedures were carried out on adult male Df(h22q11)/+ mice and their Wt littermates (age 10–13 weeks) obtained from Taconics Biosciences. A minimum number of animals was selected based on previous experience with the different paradigms. Animals were randomly chosen based on ear markings and later assigned to the correct genotype. Experimental protocols were in accordance with National (JO 887–848) and European (86/609/EEC) legislation regarding animal experimentation.

### Behavioral assays

Groups of animals (7–10 per genotype) were habituated prior to the behavioral testing by an experimenter, blind to their genotype. Tests were conducted to observe and analyze cognitive and social deficits related to schizophrenia. Animals that refused to learn the tasks after multiple trials were removed from analysis.

Supplementary information presents a complete description of all behavioral tasks.

#### Gambling task

Experiments were conducted in a four arm maze following the protocol described in Pittaras et al.^28^ where depending on the choice of the arm, the mouse receive up to 460/370 food pellets in two advantageous arms but only 220/225 pellets in the remaining two disadvantageous arms. The decision-making ability of mice was interpreted based on the % advantageous choices over 100 trials. Mice were divided into two groups using K-means clustering based on the average preference of the animals in the last three sessions to ensure the stability of choice: “risky”: mice that continued to choose the disadvantageous arms for the higher initial reward despite the high probability of receiving a penalty in the end or “safe”: mice that learnt quickly to choose one of the advantageous arms over the disadvantageous ones. The preference for % advantageous arm was compared to chance (50%).

#### Attentional set shifting task

Mice were tested on successive discrimination tasks^29,30^ where the rules in relation to the correct combination of the stimuli: digging medium and odor, were changed to measure the flexibility in attention. The number of trials required for each mouse to reach criterion (six consecutive correct trials) in every task were recorded for analysis. The combinations of correct and incorrect odor and digging medias used in the tasks are given in Supplementary Table S1.

#### Y-maze

Mice were tested on spontaneous and delayed alternations^31,32^. Exploration in all three arms of the Y-maze was performed either directly (spontaneous alternations) or after a 1 h delay from an initial training phase, where one arm of the maze was blocked (delayed alternation). Spontaneous and delayed alternation (%) were calculated as the number of entries in all three arms divided by the total number of entries in the last 5 min of the 10 min test phase, to avoid any effect of anxiety during the beginning of the test. The analysis was automated by the video tracking software, SMART (Bioseb, France).

#### Object in place task, temporal order task, and object location task (see Supplementary Methods)

In all tasks^33^, the positions of the objects were counterbalanced between mice. The discrimination ratio (DR) was calculated as the difference in time spent on the objects that were moved in time or location as compared with the time spent on the untouched objects relative to the total amount of time spent in exploration.

#### Prepulse inhibition (PPI)

The percentage of PPI^34^ induced by each prepulse intensity was calculated as 100[(SP − SPP)/SP], with SP being the average startle amplitude after the startle pulses alone and SPP being the average startle response after the combination of a certain prepulse intensity and the startle pulse.

#### Social interaction task

We investigated social repertoire, including contact types and their dynamics in freely interacting animals. Social contacts between a previously isolated host mouse (IH) and a gender and age-matched group-housed visitor mouse (V) were analysed offline using Mice Profiler software^35^. As reported earlier^36,37^, we recorded interactions for 8 min and evaluated the first 4 min of interaction.

#### Modified open field task

Mice were assessed for overall locomotion and anxiety to sudden light in a modified open field task. We followed the protocol described for rats^38^, and used a 12 min procedure. Locomotor activity was estimated based on infrared beam breaks for 8 min in a dark room followed by the sudden onset of bright light for 4 min. Anxiety-related defensive responding was estimated as the difference in distance traveled within the first minute of the light. In addition, the time spent in the zone farthest from the lamp was considered as dark preference.

### Electrophysiological experiments

#### In vivo recording of H-PFC LTP

H-PFC field potentials were recorded following the protocol described in Tripathi et al.^20^ in Df(h22q11)/+ and Wt mice (*n* = 7 each). High frequency stimulation (HFS) of the ventral hippocampus (vCA1/subiculum) consisting of two series of ten high frequency trains (250 Hz; 200 ms), was applied to induce LTP in the mPFC after 20 min of stable baseline recordings. Increasing series of stimulus intensities (100–800 μA) were used to generate input/output (I/O) curves, and the stimulation intensity which gave 80% of the maximal evoked response from Prl was chosen for baseline recordings. Local field potentials (LFPs) in the PFC were recorded for 60 min after HFS and analyzed offline using A/Dvance software. Data are expressed as a percentage change of the mean response over baseline.

#### Acute stress protocol

Animals (*n* = 7 each) were subjected to acute stress following the previously described protocol^20^. Briefly, the mice were placed on an unsteady platform (20 cm × 20 cm), 1 m above ground for 30 min in front of a bright light (1500 lux). Under these conditions, mice show freezing behavior. Blood collected from the distal end of the tail just prior to and immediately after the stress exposure was used to determine corticosterone levels using an ELISA kit. The animals were then anaesthetized and LTP induced within 90 min of completion of the stress protocol. Clozapine (Novartis) dissolved in 0.9% NaCl was administered (i.p.) at the dose of 3 mg/kg, 10 min before starting the baseline recordings.

#### Statistical analysis

All behavioral data were first analyzed for normal distribution using Shapiro Wilk’s test and then statistically compared using one-way ANOVA (for normally distributed data) or Mann–Whitney nonparametric tests (in case of non-Gaussian distribution). In case of gambling task, PPI and attention task, two-way repeated measures ANOVA was used to look at interaction effect of the variations. Electrophysiology data were statistically analyzed using repeated measures two-way ANOVA (Prism, GraphPad). For all results, *p* < 0.05 was considered as significant.

### Immunohistochemical analysis of neuronal subtypes

Coronal mice brain sections (*n* = 5 each) were immunoreacted with Anti-NeuN (ABN 90; Merck-Millipore) and anti-parvalbumin (anti-PV; PV 27; Swant) to visualize the total number of neurons and the percentage of PV inhibitory interneurons, respectively. PFC sections were photographed at low magnification on Nikon Eclipse E600 microscope and the borders of mPFC were demarcated using the Franklin and Paxinos’s atlas^39^. For quantification purposes, all PV interneurons in layers 5/6 of mPFC were counted using the cell counter plugin of Image J software, while NeuN labeled neurons were estimated using the watershed algorithm and “analyze particles” tool of the same software.

## Results

### Df(h22q11)/+ mice show cognitive deficits, reduced PPI, and altered social interaction

Df(h22q11)/+ mice showed reduced sensorimotor gating, selective attention deficits, impaired emotional decision-making as well as diminished social interactions.

#### Df(h22q11)/+ mice exhibited disparate decision-making choices in the gambling task

**Fig. 1:**
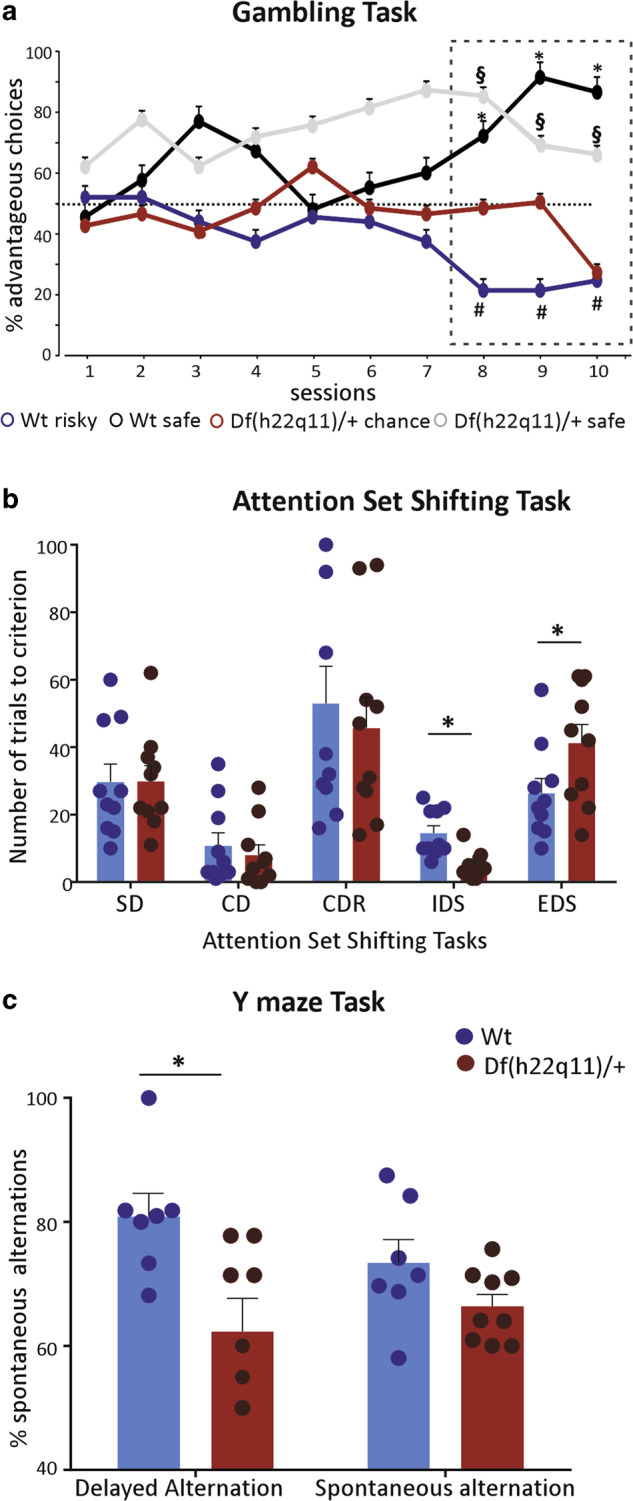
Behavioral phenotype of Df(h22q11)/+ mice and wild-type (Wt) littermates in PFC-dependent tasks. **a** Gambling task comparing differences in probabilistic learning based decision making under uncertainty in Wt and Df(h22q11)/+ mice. Graph represents the average percentage of advantageous arms ± SEM of different subgroups of Wt and Df(h22q11)/+ mice during the gambling task over 10 days. The animals were divided into safe or risky groups based on their choice for advantageous over chance level (denoted by dashed lines). The box denotes performance of animals over the last 3 days that were used for statistical analysis. Statistical difference from chance level: *p < 0.05 for Wt “safe”; ^#^*p* < 0.05 for Wt “risky”; ^§^*p* < 0.05 for Df(h22q11)/+ “safe” mice; two-way repeated measures ANOVA. **b** Attention set shifting task showing poor cognitive flexibility of Df(h22q11)/+ mice in the extra dimensional set shifting (EDS) task. Mixed bars/scatter plots represent the number of trials to criterion of six consecutive correct trials (±SEM) for each discrimination task for Wt and Df(h22q11)/+ mice during the attention set shifting paradigm (*p < 0.05), two-way ANOVA). The discrimination tasks used were: SD simple discrimination, CD compound discrimination, CDR compound discrimination reversal, IDS intradimensional set shifting, EDS extradimensional set shifting task. **c** Y maze task: impaired short-term spatial memory in Df(h22q11)/+ mice. Mixed bars/scatter plots represent the number of alternate arm entries relative to the total number of arm entries after a delay of 1 h (left) and the number of spontaneous alternate arm entries relative to the total number of arm entries (right) for both Wt and Df(h22q11)/+ mice (*p < 0.05), one-way ANOVA. All error bars indicate SEM.

Both Wt and Df(h22q11)/+ mice explored each of the individual arms at chance preference level in the beginning of the gambling task (Fig. 1a; Supplementary Fig. 1A). Toward the end of the sessions, 4/10 Wt mice showed a significantly higher preference for the advantageous arms (sessions 8–10: *p* < 0.005; *F*_(3,12)_ = 8.829) and were considered “safe”, while six animals could be categorized as “risky” as they chose the disadvantageous arms more frequently (sessions 8–10: *p* < 0.005; *F*_(3,20)_ = 5.946). In contrast the Df(h22q11)/+ mice could be divided into two equal groups. The “safe” animals showed a consistent significant preference for the advantageous arms during the task (sessions 8–10: *p* < 0.005; *F*_(3,16)_ = 11.68). The other subgroup, however, continued the exploration at chance level throughout the duration of the task and only in the last session showed a tendency toward risky behavior (sessions 8–9: *p* = 0.99, session 10: *p* = 0.14; *F*_(3,16)_ = 1.957). These mice were labeled as “chance” mice. None of the Df(h22q11)/+ mice could be categorized as “risky” mice. There was also a significant interaction subgroup × time (*p* = 0.0023; *F*_(27,144)_ = 2.136, repeated measures ANOVA). These results indicate a difference in probabilistic learning by Df(h22q11)/+ mice as compared with the Wt mice under uncertainty.

#### Df(h22q11)/+ mice demonstrated poor cognitive flexibility in the attentional set-shifting task

All mice readily learnt to dig in the bowls for food and spent increasingly less time in exploration of the surrounding box during the trials. During the simple discrimination (SD) task (Fig. 1b), both Wt and Df(h22q11)/+ mice learnt to discriminate between baited and unbaited bowls taking on average 30 ± 5 trials to reach criterion. In the compound discrimination (CD) task, the performance of either genotype was not affected by the addition of another irrelevant dimension (Wt: 10.8 ± 3.6 vs. Df(h22q11)/+: 8 ± 2.8). Reversal learning (CDR) was a much more complicated task to learn and all animals required the maximum number of trials to reach criterion. However, again no significant difference was observed in the average number of trials necessary to learn the task (Wt: 52.9 ± 10.9 vs. Df(h22q11)/+: 45.7 ± 8.6). During the introduction of new digging media and odors in intradimensional set shifting task, the Df(h22q11)/+ mice required significantly less number of trials to reach criterion (Wt: 14.5 ± 2.06 vs. Df(h22q11)/+: 4.8 ± 1.19; Mann–Whitney *U* = 8.0, *p* = 0.0006, two tailed). The performance was however reversed in case of the extradimensional set shifting task and the Df(h22q11)/+ mice performed significantly worse requiring an average of 41.2 ± 5.3 trials to reach the criterion as compared with 26.3 ± 4.2 trials for the Wt mice (*p* = 0.04; *F*_(1,18)_ = 4.44, one-way ANOVA).

#### Df(h22q11)/+ mice show impaired short-term spatial memory in the Y-maze task

Spatial recognition memory based on novelty exploration was investigated during a two-trial delayed alternation Y-maze task. The Df(h22q11)/+ mice showed a significant decrease in delayed alternations (Fig. 1c; Wt: 80.9 ± 3.4 vs. Df(h22q11/ + : 61.0 ± 5.5, *p* = 0.01; *F*_(1,14)_ = 7.95), although there was no significant difference in the total number of arm entries (Wt: 29.1 ± 2.6 vs. Df(h22q11)/+ mice: 36.8 ± 4.1, *p* = 0.16; *F*_(1,14)_ = 2.22) or in the performance of spontaneous alternations (Wt: 73.4 ± 3.5 vs. Df(h22q11)/+: 66.3 ± 1.8, *p* = 0.09; *F*_(1,14)_ = 3.17, one-way ANOVA) when the animals were allowed to freely explore the Y-maze.

#### Df(h22q11)/+ mice exhibit impaired associative recognition memory

##### Object in place task

**Fig. 2:**
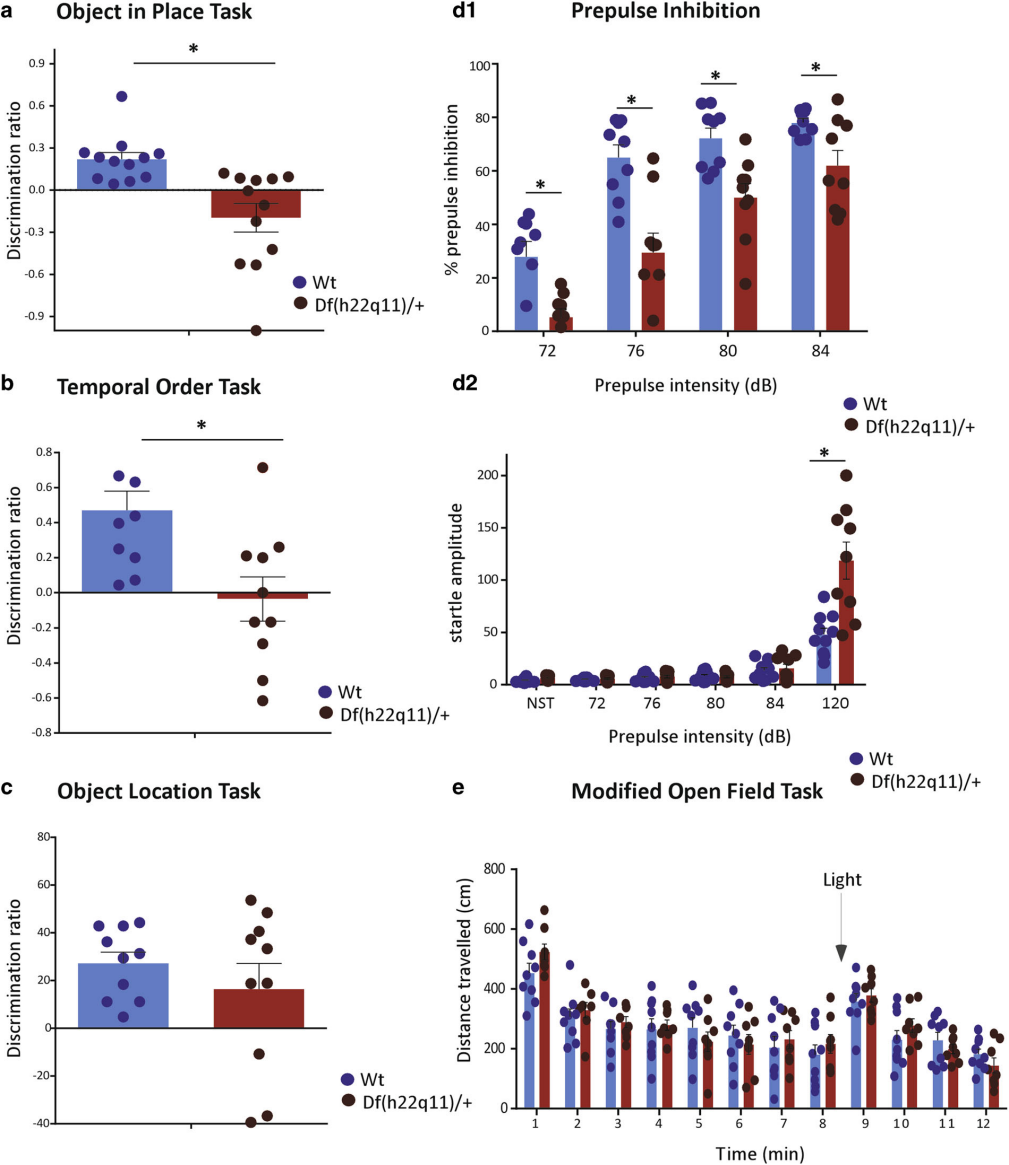
Behavioral phenotype of Df(h22q11)/+ mice and wild-type (Wt) littermates in object recognition memory tasks and schizophrenia-relevant behavioral tasks. **a** Object-in-place task: Df(h22q11)/+ mice showed a preferential exploration of the objects that remained in the old location, while all Df(h22q11)/+ and Wt mice spent similar time to explore the four identical objects. The discrimination ratio (DR) represented the time spent on exploring the displaced object minus the time spent exploring the objects in the original position divided by the total exploration time. **b** Temporal order task: Df(h22q11)/+ mice did not show any temporal differentiation and explored objects from both trial phases equally long. The DR represented the time spent in exploring the objects presented during training phase 1 minus the time spent during training phase 2 divided by the total exploration time. **c** Object location task: Df(h22q11)/+ mice gave a discrimination ratio not significantly different from Wt when exploring the displaced object. The DR represented the time spent exploring the object in the new location minus the time spent in exploring the objects in the old location divided by the total exploration time. **d** Prepulse inhibition (PPI). **d1** Df(h22q11)/+ mice showed decreased PPI. The reduction in the percentage of PPI (±SEM) is observed in Df(h22q11)/+ mice at all startle intensities tested. **d2** Compares the acoustic startle amplitude in Wt and Df(h22q11)/+ mice. **e** Locomotor activity investigated in a modified open field task showed no significant difference between Df(h22q11)/+ mice and Wt littermates. The graph represents total locomotor behavior for mice recorded for 12 min. A decrease in locomotor activity till minute 8 was observed when the shining of light caused a sudden peak in locomotor activity for both genotypes. All histograms compare the mean performance, while the scatter plots represent individual performance of Wt and Df(h22q11)/+ mice in the different behavioral paradigms. All error bars indicate S.E.M. *p < 0.05, one-way ANOVA and two-way ANOVA (PPI solely).

Wt mice spent significantly more time in exploring objects where position had been swapped as compared to unmoved objects (Fig. 2a; Wt: 61 ± 2.3 vs. Df(h22q11)/+: 40.2 ± 4.9, Mann–Whitney *U* = 0, *p* < 0.0001, two tailed). In contrast, although the total exploration time within the box was comparable, the Df(h22q11)/+ mice showed a preferential exploration of the objects that remained in the old location in the test phase relative to the objects that had swapped position with a significant difference in DR (Wt: 0.22 ± 0.04 vs. Df(h22q11)/+: −0.19 ± 0.09, Mann–Whitney *U* = 15, *p* = 0.0005, two tailed).

##### Temporal order task

Wt animals spent significantly more time exploring the object from the first trial period (Fig. 2b; 73.5 ± 5.2 s) as compared with the more recent object of trial phase 2 (% exploration = 26.5 ± 5.2, *p* = 1.08 × 10^−5^; *F*_(1,18)_ = 36.27). In contrast, Df(h22q11)/+ mice did not show any temporal differentiation and explored objects from both trial phases equally long (% exploration 1st trial object = 48.2 ± 5.1 vs. % exploration 2nd trial object = 51.8 ± 7.6). Performance analysis of the two groups based on DR revealed a significant deficit in temporal organization for Df(h22q11)/+ mice (Wt: 0.47 ± 0.1 vs. DF(h22q11)/+: −0.03 ± 0.11, *p* = 0.007; *F*_(1,18)_ = 9.13).

##### Object location task

Both Wt and Df(h22q11)/+ mice performed similarly in this task. No significant difference was observed in DR (Fig. 2c; Wt: 27.2 ± 4.4 vs. Df(h22q11)/+: 16.4 ± 10.1) or in the total amount of exploration time (*p* = 0.37; *F*_(1,18)_ = 0.85).

#### Df(h22q11)/+ mice exhibit poor prepulse inhibition

Sensorimotor gating was evaluated using the PPI paradigm. The amplitude of the startle response was significantly higher in case of the Df(h22q11)/+ mice (Fig. 2d2; *p* = 0.001; *F*_(1,18)_ = 15.33), whereas percentage PPI in Df(h22q11)/+ mice was reduced (Mean PPI: *p* = 0.0003; *F*_(1,16)_ = 21.912). The reduction was seen at all prepulse intensities tested (Fig. 2d1) and no significant interaction was found between the prepulse intensity and genotype (*p* = 0.1; *F*_(3,24)_ = 2.321, two-way ANOVA repeated measures).

#### Df(h22q11)/+ mice exhibit impairment in social contacts

**Fig. 3:**
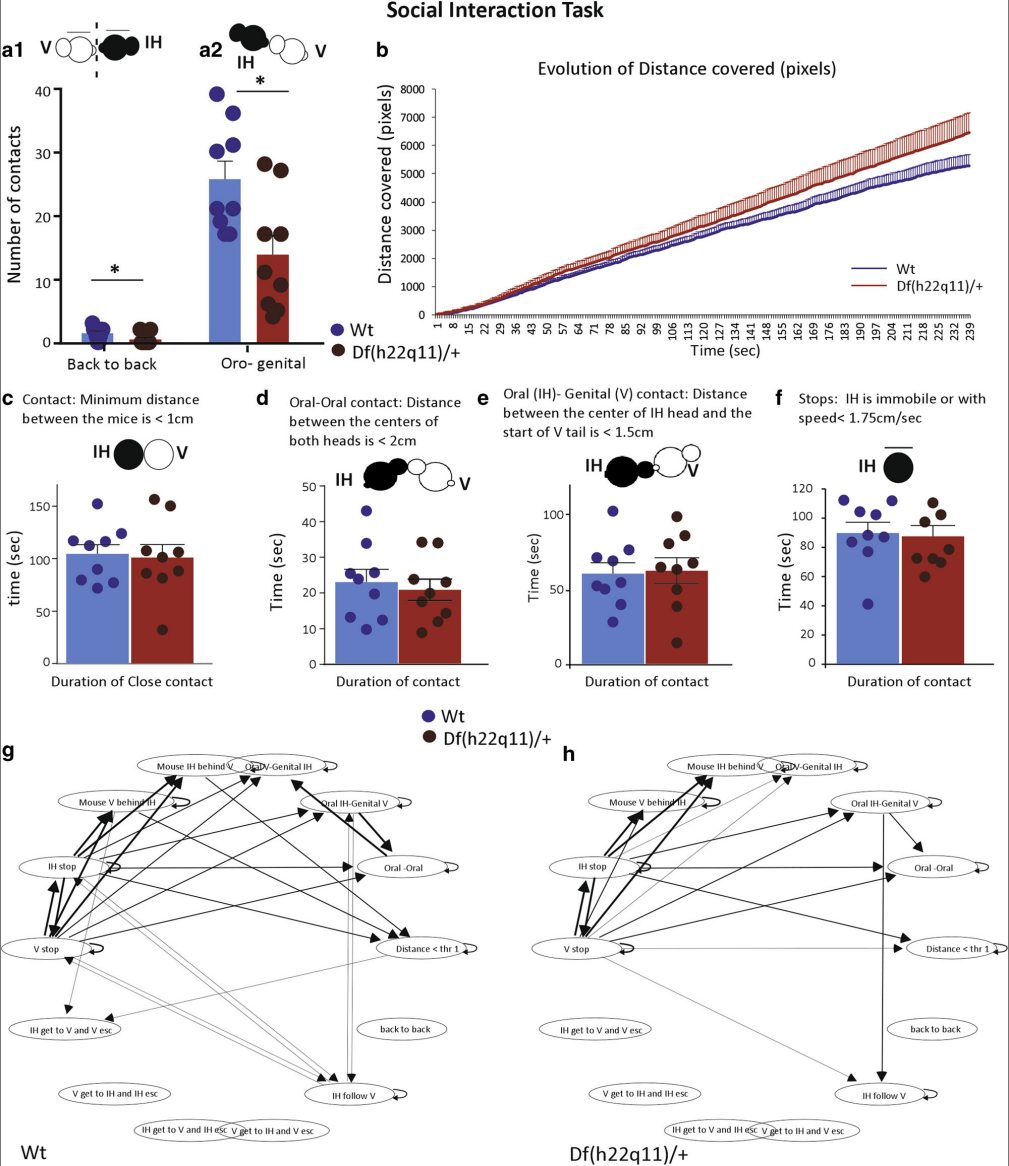
Behavioral phenotype of Df(h22q11)/+ mice and wild-type (Wt) littermates in the social interaction task. **a** Df(h22q11)/+ mice presented impaired social contacts. The number of back-to-back (**a1**) and oro(V)-genital(IH) contacts (**a2**) in social interaction task differ significantly between Wt and Df(h22q11)/+ mice during 4 min of analysis. **b** Represents the difference in the evolution of the distance covered by both Wt (blue) and Df(h22q11)/+ mice (red) across sessions during 4 min of analysis indicating that Df(h22q11)/+ mice covered more distance toward the end of the analysis time. **c**–**e** Bar graphs represent the difference between duration of the various contact events analysed in the present study. Both Wt and Df(h22q11)/+ mice displayed comparable duration of close contact (**c**), oro–oral contacts (**d**), and oro–genital contacts (**e**) between the isolated host (IH) and the visitor mouse (V). **f** The bar graphs represent the number and duration of stop events by the isolated host during 4 min of analysis. **g**, **h** Comparison of the decision trees based on the performance of Wt and Df(h22q11)/+mice in the social interaction task. Thickness of arrow indicates the probability of transition from one behavioral event to the other (i.e., the larger the arrow, the higher the probability), whilst the number of connections suggest the number of behavioral options available to that genotype of mice. Wt and Df(h22q11)/+ animals are figured with blue and red bars, respectively. Individual data are represented in each graph by a black dot. Isolated host (IH) is represented with black circle and Visitor (V) mouse with white circle on each inset depicting the social event analysed. All values are represented as mean ± S.E.M. *p < 0.05, Mann–Whitney.

Social interactions are complex adaptive behaviors integrating numerous emotional and motivational choices to initiate and execute actions. Df(h22q11)/+ and Wt mice exhibited a similar social repertoire (Fig. 3). Number and duration of close and oro–oral contacts were not significantly different between genotypes (Fig. 3c, d respectively for contact duration). Neither events demonstrating relative positions (IH behind V and V behind IH) nor dynamic events initiated by the IH or the V mouse (IH or V approached, followed, and made contact or IH or V escaped after contact), showed any statistical changes for duration and number. Regarding stops events by the V or the IH mouse, no significant genotype effect was found (Fig. 3f for duration of IH stops, data not shown for numbers and V stops).

However, Df(h22q11)/+ mice displayed lower number (Fig. 3a1; Mann–Whitney *U* = 19, *p* = 0.038, two tailed) and duration (data not shown) of back-to-back contacts. In addition, for oro–genital contacts, behavior differed between groups depending of which animal made oral or genital contacts. While IH oral contact with genital V mouse was similar between genotype (Fig. 3e for duration; not shown for number), V mouse made significantly less oro–genital contacts with Df(h22q11)/+ mice as compared with Wt mice, (contact number: Mann–Whitney *U* = 16, *p* = 0.01, two tailed), suggesting a lower tolerance of Df(h22q11)/+ mice to V initiatives (Fig. 3a2).

Although the total distance traveled by the Df(h22q11)/+ and Wt mice during the complete session did not differ (data not shown), both groups did not evolve similarly across session. Repeated measure ANOVA showed a clear time effect (*p* < 0.0001; *F*_(15,238)_ = 184.14) and an interaction effect time × genotype (*p* < 0.0001; *F*_(15,238)_ = 1.597) with the Df(h22q11)/+ mice covering more distance toward the end of the session, suggesting a potential habituation deficit (Fig. 3b).

Despite a similar social behavior repertoire for Wt and Df(h22q11)/+ mice, transitions between events were not evenly distributed between the two groups. Indeed, decision trees based on relationship probability between two social behavior events revealed a simpler decision tree in Df(h22q11)/+ mice as compared with Wt mice, (Fig. 3g, h respectively), suggesting lack of strong relationship between behavioral sequences in Df(h22q11)/+ mice (i.e., the probability that one event follows a specific other one is very low, consequently a number of behavioral options are larger).

#### Df(h22q11)/+ mice show no difference in locomotion or anxiety-like behavior

There was no significant genotype difference in the total amount of distance traveled before or after exposure to sudden light (Fig. 2e, *p* = 0.99; *F*_(1,22)_ = 0.00015). Neither quantification of rearing behavior nor distance traveled/time spent in the zone farthest away from light showed any difference indicating similar baseline anxiety and dark preference between Df(h22q11)/+ and Wt mice.

#### Df(h22q11)/+ mice exhibit impaired H-PFC LTP

**Fig. 4:**
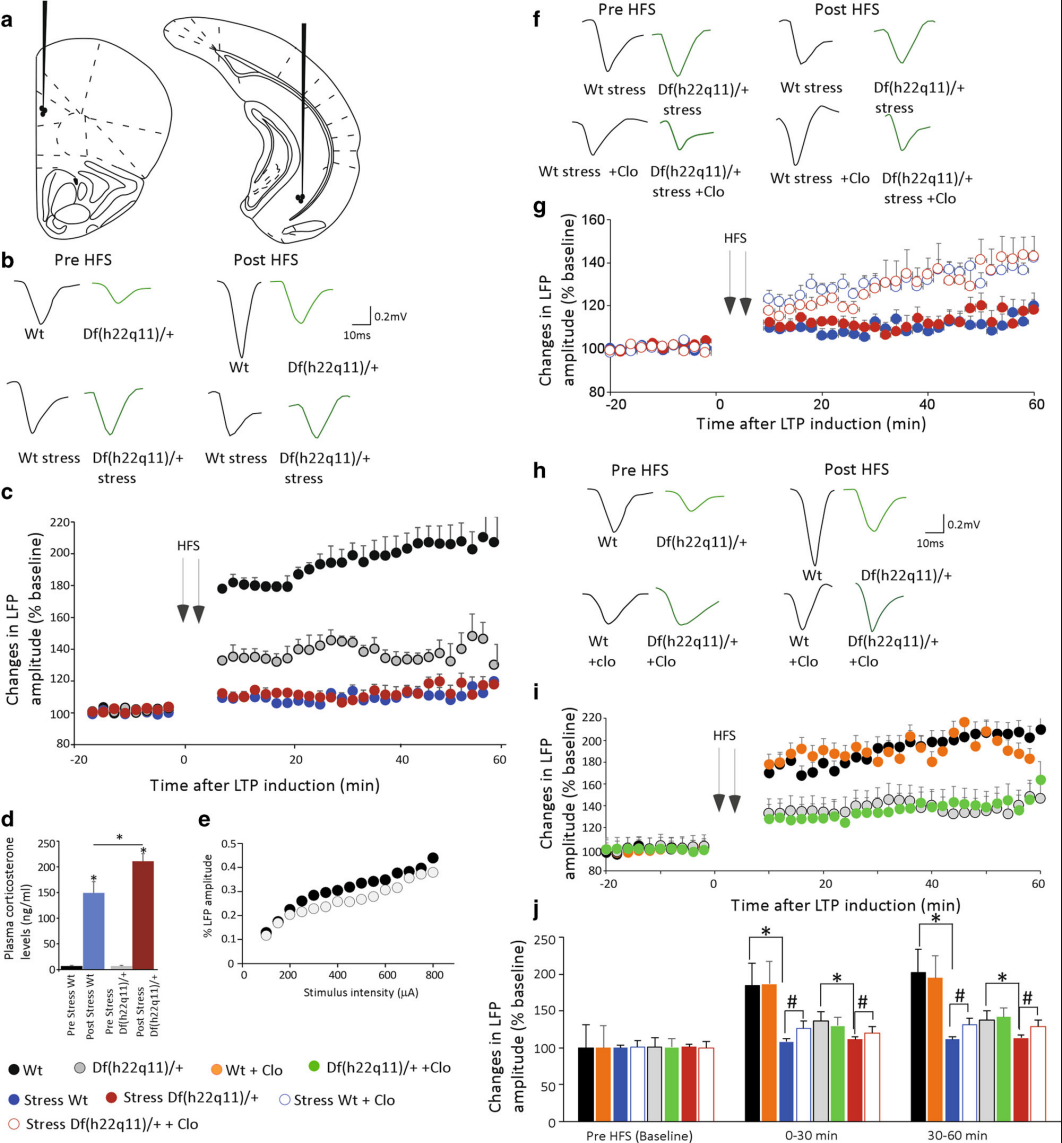
Electrophysiological in vivo characterization of H-PFC synaptic plasticity in Df(h22q11)/+ mice. **a** Schematic representation of the position of the recording electrode in the Prl of the mPFC and the stimulating electrode in the ventral hippocampus. **b** Representative average waveform (4) of evoked local field potentials (LFPs) taken at pre-HFS and 10 min post-HFS times in nonstressed and stressed Wt and Df(h22q11)/+ mice (green). **c** Df(h22q11)/+ mice (gray) are severely impaired in H-PFC LTP compared to Wt mice (black) (two-way ANOVA, repeated measures, *p* < 0.0001 for genotype and for time × genotype interaction; *n* = 9 per group). Acute stress produced long-lasting block of the H-PFC LTP in both Wt (blue) and Df(h22q11)/+ mice (red) (three-way ANOVA, repeated measures, *p* < 0.0001 for genotype and stress effect; *n* = 7 per group). **d** Exposure to platform stress resulted in an increase in plasma corticosterone levels in both genotypes. Df(h22q11)/+ mice showed significantly higher corticosterone levels relative to Wt mice immediately after stress. All error bars indicate S.E.M. *p < 0.05, one-way ANOVA. **e** Input/output response curves of H-PFC LFPs in Wt and Df(h22q11)/+ mice. No significant difference was observed between the two groups, one-way ANOVA, *n* = 5 per group. **f** Representative average waveform (4) of H-PFC LFPs taken at pre-HFS and 10 min post-HFS times in Wt and Df(h22q11)/+ mice (green) after stress (green) and after stress and clozapine injection respectively. **g** Effects of clozapine on stress-related alterations in H-PFC LTP. Df(h22q11)/+ mice (red open circles) showed a full recovery of stress-impaired LFP amplitude 60 min after HFS, while Wt mice (blue open circles) showed a partial recovery after injection of clozapine (3 mg/kg; i.p.) (three-way ANOVA, repeated measures, *p* < 0.0001 for time, genotype, and clozapine effect; *n* = 7 per group). **h** Representative average waveform (4) of H-PFC LFPs taken at pre-HFS and 10 min post-HFS times in Wt (black) and Df(h22q11)/+ mice (green) under control conditions and after clozapine injection respectively. **i** Effects of clozapine on H-PFC LTP in unstressed mice. In control, nonstressed animals, injection of clozapine (3 mg/kg; i.p.) caused no change in H-PFC LTP in either Wt (orange vs. black circles) or Df(h22q11)/+ mice (green vs. gray circles). **j** Comparison of LFP amplitudes during baseline, 30 and 60 min after HFS in Wt mice and Df(h22q11)/+ mice under control conditions, stress, and after clozapine injection. All error bars indicate S.E.M. *p < 0.05 for control vs. stress; ^#^*p* < 0.05 for stress vs. stress + Clo mice.

The magnitude of H-PFC LTP was markedly reduced in the Df(h22q11)/+ mice during the whole recording time (Fig. 4). The HFS-induced percentage increase in LFP amplitude analyzed for 60 min (*T*_60_) relative to the baseline (*T*_0_) was significantly reduced for Df(h22q11)/+ mice relative to Wt mice (Fig. 4c, Df(h22q11)/+ *T*_60_: 137.7 ± 1.0 vs. Wt *T*_60_: 195.0 ± 2.1; two-way repeated measures ANOVA; *p* < 0.0001; *F*_(1,12)_ = 48.46 for genotype and *p* < 0.0001; *F*_(26,312)_ = 3.06 for time × genotype interaction). Input–output response curves for Wt and Df(h22q11)/+ mice show no significant difference between the two groups (Fig. 4e, *p* = 0.18; *F*_(1,28)_ = 1.82, one-way ANOVA) which indicate that the LTP impairment in Df(h22q11)/+ mice is not due to smaller basal synaptic responses.

#### H-PFC LTP is significantly reduced by stress in both Wt and Df(h22q11)/+ mice

Exposure to acute platform stress prior to LTP induction led to a significant decrease in the LFP amplitude in both Wt and Df(h22q11)/+ mice recorded up to 60 min after HFS (Fig. 4c). Stress led to a very marked decrease in LTP in both control and Df(h22q11)/+ mice; LTP was almost abolished by this protocol. There was a clear genotype (*p* < 0.0001; *F*_(1,26)_ = 845.14) and stress (*p* < 0.0001; *F*_(1,26)_ = 660.27) effect on LFP amplitude throughout the recording period after HFS (three-way ANOVA, repeated measures). In case of stressed Wt mice, HFS led to a significant 43% decrease in LFP amplitude (*T*_60_ = 110.2 ± 0.6). A robust significant increase in plasma corticosterone levels was observed after the 30 min period of stress in both Wt (Wt_post_: 148.7 ± 21.9 ng/ml vs. Wt_pre_: 6.8 ± 1.2 ng/ml; *p* = 1.6 × 10^−6^; *F*_(1,22)_ = 41.74) and Df(h22q11)/+ mice (Df(h22q11)/+ _post_: 210.5 ± 14.6 ng/ml vs. Df(h22q11)/+ _pre_: 6.9 ± 1.07 ng/ml; *p* = 2.29 × 10^−12^; *F*_(1,22)_ = 192.82). Df(h22q11)/+ mice demonstrated significantly higher poststress corticosterone levels relative to Wt mice (Fig. 4d; *p* = 0.03; *F*_(1,22)_ = 5.49, one-way ANOVA).

#### Clozapine prevents stress-induced disruption of LTP in Df(h22q11)/+ mice

The potential of clozapine to prevent the effects of stress on LTP was tested in Wt and Df(h22q11)/+ mice. Clozapine, when administered after stress, led to a complete vs. partial recovery of stress-induced impairment of H-PFC LTP in Df(h22q11)/+ vs. Wt mice (Fig. 4g). LTP in stressed mice after clozapine was comparable between Wt and Df(h22q11)/+ mice (Wt stress + clo *T*_60_: 131.1 ± 0.8 vs. Df(h22q11)/+ stress + Clo *T*_60_: 129.4 ± 0.9, *p* = 0.86, *F*_(1,50)_ = 0.03). Most significantly, clozapine led to a complete restoration of LTP to control levels in stressed Df(h22q11)/+ mice, by 60 min (Df(h22q11)/+ mice stress + clo *T*_30–60_: 135.9 ± 1.3 vs. Df(h22q11)/+ *T*_30–60_: 138.3 ± 0.9, *p* = 0.1, *F*_(1,30)_ = 2.86). In contrast in Wt there was a significant but partial recovery of LTP after clozapine injection during the entire recording period (Wt stress T_60_: 110.2 ± 0.6 vs. Wt stress + clo *T*_60_: 131.1 ± 0.8, *p* < 0.001; *F*_(1,50)_ = 99.99; Wt *T*_60_: 195.0 ± 2.1 vs. Wt stress + clo *T*_60_: 131.1 ± 0.8, *p* < 0.01; *F*_(1,50)_ = 508.65, one-way ANOVA). In case of nonstressed mice, clozapine injection caused no significant effect on LTP amplitude in either Wt or Df(h22q11)/+ mice (Wt: *p* = 0.31, *F*(1,52) = 1.49; Df(h22q11)/+: *p* = 0.42, *F*_(1,52)_ = 0.67, one-way ANOVA).

#### Reduction in PV interneurons in the mPFC of Df(h22q11)/+ mice

**Fig. 5:**
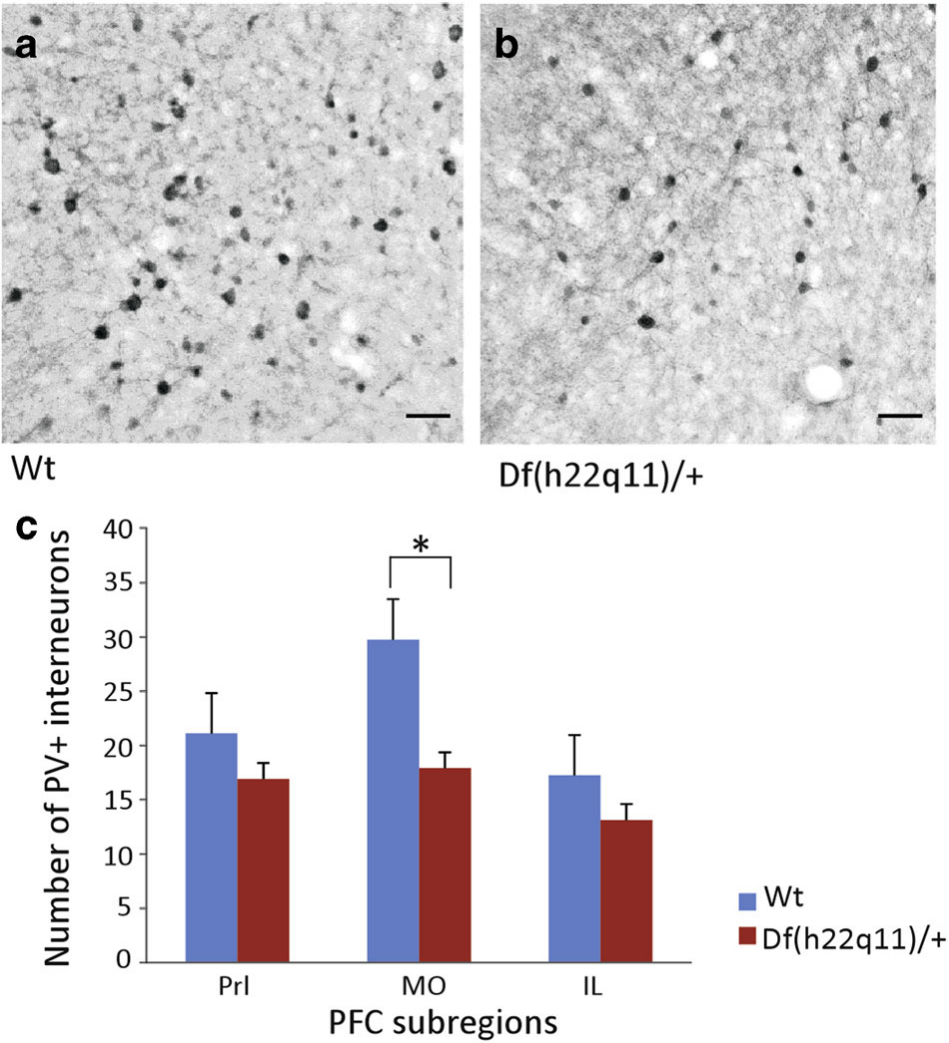
Quantification of parvalbumin interneurons in the mPFC in Df(h22q11)/+ mice and wild-type (Wt) littermates. **a**, **b** The photographs are representative of the parvalbumin (PV)+ interneurons within the MO subregion of mPFC in Wt (**a**) vs. Df(h22q11)/+ mice (**b**). **c** The bar graphs show a significant decrease in the PV+ interneuron population in MO as compared with Prl or IL subregions of mPFC in Df(h22q11)/+ mice relative to Wt mice. All error bars indicate S.E.M. *p < 0.05, one-way ANOVA. (*n* = 5 per group) Scale bar = 50 μm.

We observed a significant decrease in PV interneurons in the mPFC of Df(h22q11)/+ mice (Fig. 5, Wt: 68.2 ± 6.6 vs. Df(h22q11)/+: 48.0 ± 4.5, *p* = 0.003; *F*_(1,8)_ = 16.96, one-way ANOVA). The reduction in the number of interneurons was also analyzed with respect to the total number of neurons. In all subregions of the mPFC, there was <10% difference in the total number of neurons between genotype suggesting a genuine region-specific decrease in PV interneurons, independent of the total number of neurons (WT _% of PV/NeuN_ = 6.13% vs. Df(h22q11)/+ _% of PV/NeuN_ = 3.094%).

## Discussion

The de novo CNV, 22q11.2 microdeletion, is one of the highest genetic risk factor for schizophrenia^40^. Cognitive deficits as well as functional disconnections between regions of the corticolimbic circuit are core pathological features in schizophrenia^16,41–44^. In the present study, we demonstrate that the Df(h22q11)/+ mice show quantifiable deficits in synaptic plasticity in the H-mPFC circuit. The role of this pathway is to link hippocampal spatial and temporal context to PFC linked to amygdala emotive outputs^45^, so baseline reduction of plasticity in the H-PFC may be responsible for cognitive and social deficits, as has been recently shown for memory deficits in the aged^46^. The impact of stress would further exacerbate this inhibition. Furthermore, these mice showed increased corticosterone levels, which is a feature of 22q11.2 deletion syndrome (22q11.2DS). Jacobson et al.^47^ proposed that increased glucocorticoid secretion is causal to social impairment, in conditions of stress in children. In support, Armando et al.^48^ have investigated life stress, pituitary function, and volume in subjects with 22q11.2DS, concluding that stress and coping are central in the pathogenesis of psychosis. We have shown in mice and rats that plasticity in the H-PFC circuit is impaired by stress, and corticosterone, and that this circuit may be a weak link in psychiatric disorders (see review^45^). Thus, stress and its effects in the Df(h22q11)/+ mouse model may be crucial for screening therapeutic approaches from a neural circuit perspective^49^.

The Df(h22q11)/+ mice showed robust cognitive deficits in several PFC dependent tasks. In attentional set shifting task, Df(h22q11)/+ mice show attentional deficits at the extradimensional shift abilities with an unaffected reversal learning. Df(h22q11)/+ mice are also deficient in spatial recognition memory on the delayed alternation Y-maze task, in the object-in-place, and temporal order recognition memory tasks but not in the object location memory. In the rodent version of the Iowa gambling task (IGT), where mice made arm choices based on initial and long term reward/penalty assessment, Df(h22q11)/+ mice show a clear difference in the decision-making approach. While Wt mice could clearly be segregated into animals that preferred long term high rewards (safe) or initial high reward but overall low gain (risky), there was a subset of Df(h22q11)/+ mice which failed to learn any strategy. Upon choosing an arm, they persevered to enter the same arm throughout that session. Those cognitive tasks are classically used to measure working memory, attentional set shifting, behavioral flexibility and decision-making, and their optimal performance depends on the integrity of the PFC and PFC circuitry^50–52^. A clear deterioration of decision-making performance on IGT is measured in patients with schizophrenia^53^. The perseverative behavior shown in Df(h22q11))/+ mice has also been recorded in schizophrenia patients. More interestingly, deficits in attention shifting and set maintenance with more perseverative errors (as measured by the Wisconsin Card Sorting Test) were recently reported in a longitudinal study on 22q11DS patients who developed psychotic symptoms^54^ and proposed as a key feature for identifying individuals with 22q11DS at risk for developing psychosis.

Social isolation, withdrawal, and inability of schizophrenia patients to interact adeptly in a social setting are common aspects of the disorder. Social impairments are also described as a common feature of the 22q11.2DS^55^. The PFC integrity and neurotransmission have been reported in social approach and proximity to a congener in mice^56^. While Df(h22q11)/+ mice exhibited a complete social repertoire, they show differences in social tolerance, a trend toward social aggression and a simpler decision tree based on relationship probabilities between social behaviors. This may suggest that social repertoire use is different in the Df(h22q11)/+ mice, with a more disorganized and stereotyped social behavior.

Although the Df(h22q11)/+ mice exhibit poor performance in the above-mentioned tasks, simple tasks involving the hippocampus, such as the open field and object location tasks, show no impairment. This is consistent with other 22q11 mouse models that show no deficit in locomotion or exploratory habits, anxiety levels^12^, or in simple recognition memory tasks^4^. There was also no substantial difference in simple recognition memory tasks^4^. In addition, our mouse model like other 22q11 deletion animal models^57^, showed a significant deficit in sensorimotor processing, as well as heightened startle amplitude, reinforcing the importance of chromosome 22q11 segment in the modulation of preattentional information processing.

Significant cognitive deficits were observed in several tasks known to require intact circuitry between the PFC and hippocampus such as the object in place and temporal order recognition memory and spatial recognition memory^58–60^. The two recognition memory tasks involve additional recent discrimination and integration plus association of object recognition and object location memory. Both the encoding as well as the retrieval of such information are dependent on the H-PFC and H-perirhinal cortical circuit where the encoding is known to be NMDA dependent^59^. Interestingly, Df(h22q11)/+ mice have been reported to show NMDA-related dysfunction with increased locomotion in response to NMDA antagonists^8^.

Within the H-PFC pathway, tetanic stimulation of the hippocampal outflow through the vCA1/subicular region to the PFC led to a long-lasting increase in synaptic efficacy recorded from mPFC, which was significantly reduced in Df(h22q11)/+ mice, perhaps an underlying causes of abnormal functional coupling reported as a phenotype relatively specific to schizophrenia^19^. This may well be the result of chronic mismanagement of stress, or a consequence of altered neurodevelopmental plasticity due to deficits in PV neuron recruitment as recently reported^61^. One other reason for the decrease in H-PFC plasticity could be an abnormal mesocortical dopaminergic activity affecting the dendritic spines on both pyramidal and local circuit neurons in Layer 5 and 6 of the mPFC^62,63^. Indeed, activation of D1 receptors through a modulation of NMDA receptors favors the induction of LTP at H-PFC synapses by increasing NMDAR-mediated responses in PFC^64^. Dopamine release in the PFC is thought to modulate H-PFC synchrony^58^ and tune the signal-to-noise ratio within mPFC networks^65^. In line with this hypothesis, biochemical assays have shown an increase in dopamine metabolite 3,4-dihydroxyphenylacetic acid (DOPAC) in the PFC along with aberrant NMDA functioning in Df(h22q11)/+ mice^8^, while alterations of dopamine modulation of PFC interneurons have been reported in other mouse models of the 22q11.2DS^66^.

Plasticity of the H-mPFC circuitry is particularly vulnerable to stress in mice and rats^20,21^. Increases in plasma glucocorticoids directly decrease synaptic plasticity in H-mPFC^21,67^ so the higher poststress corticosterone levels in Df(h22q11)/+ mice may be causative via modifications of PFC D1 receptor stimulation and AMPA receptor phosphorylation (mostly GluA1) affecting cognition and synaptic efficacy^68,69^. Increased PFC DOPAC and dorsal striatal GluA1 receptor levels have been found in Df(h22q11)/+ mice^8^, and a potential reduced number of D1 and/or GluA1 receptors in the PFC may also be responsible for the discrepancy in the effect of stress in the magnitude of H-PFC plasticity between Wt and Df(h22q11)/+ mice^70,71^. Corticosterone has been shown to increase GluA1 receptor mobility directly, thereby reducing levels of GluA1 in the synapses of hippocampal neurons, correlated with inhibition of LTP^72^, while reinsertion in the synapse with an antidepressant restored LTP. Thus, the dopaminergic/AMPA balance may be changed. Clinically, there is an increased risk of early-onset Parkinson’s disease associated with 22q11.2DS^73^. We previously observed that a poststress treatment of low dose clozapine protected H-PFC plasticity from stress-induced disruption by ~70%^26^. Here we demonstrate that clozapine, administered acutely after stress, so that only the effects on H-mPFC dysfunction, but not the stress itself, were modified. We did not wish to administer clozapine chronically, because as clozapine is a multireceptorial drug, it would change the impact of the acute stress, with unknown effects on H-PFC plasticity. Furthermore, in clinical practice, clozapine is administered in increasing doses so as to mitigate effects on postural hypotension. The dose of 3 mg/kg i.p. chosen yields plasma levels in mice corresponding to the human clinical plasma levels^74^. Nevertheless, clozapine only partially protected against stress in the control animals, i.e., had the same net effect in both groups. We consider this important, as if H-mPFC plasticity is already reduced by neurodevelopmental factors, then further suppression by acute stressors may be deleterious. Clozapine and other, but not all, antipsychotics have been recently shown to modulate PFC-dependent cognitive functions in patients with schizophrenia and relevant mouse models depending on the genetic background of the patients^75^. Patients with 22q11.2DS may be particularly sensitive to stress^47^ and respond to clozapine, as clozapine was the only antipsychotic to be effective in a recent case report of psychosis in a 22q11.2DS^76^. It may also be that such patients are also particularly sensitive to clozapine-induced seizures and other side effects^77–79^, including perhaps myocarditis^80^. Nevertheless, the convergence of these findings suggests the possibility of potential new therapies targeting the H-PFC circuit and this model may prove useful in screening new therapies.

In schizophrenia, cognitive deficits could be the result of altered connections between pyramidal cells in the PFC and a reduced number of GABAergic interneurons specifically those containing the calcium binding protein PV^81^. A similar trend was observed in the present study with a reduced number of PV+ interneurons in the mPFC, specifically in the MO subregion in Df(h22q11)/+ mice. Interestingly, the potential of reversing PV recruitment and dysfunction in another 22q11DS mouse model was recently shown by an antipsychotic treatment (D2 dopamine receptor antagonists) although the rescue was only seen when administered during late adolescence^61^. Whether the decrease in interneuron population is associated with alterations in the functional connectivity of these interneurons remains to be investigated.

## Conclusions

In summary, the complexity of the pathophysiology of neuropsychiatric disorders such as schizophrenia results in most animal models only indexing part of the symptom cluster. The Df(h22q11)/+ mice show a definite endophenotype resembling several of the PFC dependent impairments observed in schizophrenia patients. A partial restoration of the balance between the excitatory and inhibitory activity within the H-PFC circuit in Df(h22q11)/+ mice may reinforce the potential use of this animal model for analyzing gene–environment interaction in the pathophysiology of schizophrenia, particularly where the impact of stress and coping may be critical in 22q11.2DS. We believe this animal model could be used to further investigate the neurochemical and cytoarchitectural changes that may predispose an individual to develop schizophrenia. Manipulating the interplay of different genes within this segment of 22q11 chromosome could potentially enable us to identify the molecular markers that precipitate schizophrenia pathology and thus become targets for therapeutic advances.

## Supplementary information

Supplementary Information

Supplementary Fig 1A
