# Supplementary material for: Cognition- and circuit-based dysfunction in a mouse model of 22q11.2 microdeletion syndrome: effects of stress
Source: Transl Psychiatry. 2020 Jan 28;10:41. doi: 10.1038/s41398-020-0687-z (PMC7026063; doi:10.1038/s41398-020-0687-z)
Supplement: Supplementary file 1 — Supplementary Information [file 41398_2020_687_MOESM1_ESM.docx]

**Supplementary Information**

**Supplementary Methods**

**Animals**

All experimental procedures were carried out on adult male Df(h22q11)/+ mice and their wild type (WT) littermates (age 10-13 weeks) obtained from Taconics Biosciences. The breeding schemes of the animals are detailed in Didriksen et al., 2017^1^. Briefly all animals were bred between Wt C57BL/6J females and hemizygotic Df(h22q11)/+ males. All mice were group-housed in standard mouse cages with sawdust bedding and paper for nesting under 12h light/ dark conditions and *ad libitum* access to food and water. In case of anatomical experiments, mice were individually housed after surgery for recuperation. For all behaviour experiments, the animals were food restricted to reach 80% of their body weight. Experimental protocols were in accordance with (JO 887-848) and European (86/609/EEC) legislation regarding animal experimentation.

**Behavioral assays**

After being handled for a week, the animals (10 per genotype) underwent a battery of hippocampus and PFC dependent behavioral tests. The animals were marked with their ear markings as opposed to their genotype by a different experimenter. This allowed the experimenter conducting, assessing and quantifying the trials to be blind to the genotype of the animals.

***Gambling task***

Mice were tested on their decision making ability under uncertainty of reward and/ or punishment in the mouse gambling task. This task developed by Pittaras et al.^2^ was inspired by the Iowa gambling task. Briefly the apparatus consisted of a small 20cm X 20cm holding box and 4 transparent 40cm X 10cm X 10cm maze arms which were set up in an arc, equidistant from the holding box. The goal of the task was to get the maximum number of food pellets. However, out of the 4 arms, two were considered advantageous arms where the probability to receive a penalty was minimum while 2 were the disadvantageous arms where the conditions were reversed. The reward consisted of small quantities of food pellets (Bio Serv), while the penalty consisted in food pellets dipped in a solution of 180mM Quinine (Sigma). The advantageous arms contained an initial smaller reward of 1 pellet and then a food cup at the end of the maze containing 3 /4 food pellets. Only in 1/10 cases, the animal received a penalty in these arms. On the contrary, the disadvantageous arms had an initial higher reward of 2 food pellets but then 19/20 times the animals received a penalty of 4/ 5 quinine dipped pellets. In total, after 100 trials, over 10 days, the animals had a choice of receiving 460 pellets in one advantageous arm and 370 in the other, while in the disadvantageous arms, they received 220 or 225 pellets only.

*Food restriction*

Before beginning the task, mice were food deprived to reduce their body weight to 80% of their free feeding weight. During this week, animals were habituated to small quantity of food pellets in their home cage. Four days before beginning the testing, mice were habituated to eat the pellets in a novel environment (a 30cm X 30cm box). The last step was to finally habituate the mice to the 4 arm maze and confirm that they would eat the pellets in the maze. During the everyday test, mice were first allowed to explore the maze for 5min and given a pellet to eat in each arm. Then they were held in the waiting box while the arms were baited with either reward / penalty. The animals were allowed to choose a single arm and the choice was noted. They were then returned to the waiting box for a minimum of 10 secs before the next trial. The arms were cleaned with water between each trail and a disinfectant between each mouse. Two sessions of 10 trials each was done per mouse every day for 5 days and the side of the advantageous and disadvantageous arms was randomly chosen on the first day for each mouse and then maintained during the entire task.

*Data analysis*

The decision making ability of these mice was interpreted based on the % advantageous choices over 100 trials. Based on the number of times they chose the advantageous arms over the disadvantageous arms, mice were divided into two groups: “risky” or “safe”. The risky mice continued to choose the disadvantageous arms for the higher initial reward despite the high probability of receiving a penalty in the end, while the safe animals tend to learn quickly to choose one of the advantageous arms over the disadvantageous ones. The preference for % advantageous arm was compared to chance (50%) for each genotype and analysed using one-way ANOVA.

**Attention Set shifting task**

Mice were tested on their ability to adapt to dynamic conditions for food acquisition. They had to first identify and learn the relevant stimuli for obtaining food when multiple stimuli were presented and then modify their strategy when the current set of rules were suddenly changed ^3, 4^. Briefly the apparatus consisted of a 30cm X 30cm waiting chamber which opened into two 15cm X 15cm holding areas containing a small food cup each. There were two main stimuli, the digging media in the food cup and the odor with which the cup was laced. The reward consisted in small bits of cereal. In each successive discrimination task, the rules in relation to the correct combination of digging medium and odor were changed to measure the flexibility in attention.

*Habituation.* The animals were habituated to the experiment box for 10min for 2 days prior to the beginning of the test. Additionally, each night two food cups with different digging medium and baited with small bits of cereal were placed in the home cages to habituate the mice with different digging media and to ensure swift digging for food.

*Testing.* The animals were given 4 exploratory trials on the first day which were not terminated till the food was retrieved from one of the cups. From the fifth trial the mouse was allowed to dig in only one food cup and the trial was terminated if it started digging in the wrong food cup and an error was marked. The side for presentation of the food baited cup was changed randomly between trials. The testing continued till the animal had reached the criterion of 6 consecutive correct trials. Sometimes the testing was discontinued if the mouse showed signs of stress and ignored exploration for food. After one task was learnt, the animals instantly started on the consecutive task. As the tasks are designed to test the shift in attention, every day before beginning a new task, mice were tested to criterion on the previous task as well.

*Simple discrimination (SD) task*. The animals were presented with food cups laced with two different odors (paprika and basil), of which only one cued the reward. In case of *the compound discrimination (CD) task*, a second stimulus, the digging medium was introduced and the animals had to focus on the odor while ignoring any of the two digging media (soil and paper). The same combination of stimuli was maintained in the reversal task (CDR), however the odor cueing the reward/ food was reversed. In the *Intradimensional set shifting task* (IDS), the animals were presented with new digging media and odors, however the relevant dimension signalling reward was still one of the odors (odors: clove and cinnamon; digging media: wood chips, confetti). Finally, in the extradimensional set shifting task (EDS), rules were changed such that one of the digging media now indicated food while the odors were the irrelevant dimension of stimuli (digging media: stones, cotton; odors: cumin, thyme). The different combinations of correct and incorrect odor and digging medias used in the task are given in Table 1.

| Task | Stimuli | | Combinations | | | |
| --- | --- | --- | --- | --- | --- | --- |
|  | Relevant | Irrelevant | correct | | Incorrect | |
| Simple discrimination (SD) | Odor (O) |  | O1 |  | O2 |  |
| Compound discrimination (CD) | Odor | Digging medium (M) | O1 | M1,M2 | O2 | M1,M2 |
| Compound discrimination reversal (CDR) | Odor | Digging medium | O2 | M1,M2 | O1 | M1,M2 |
| Intradimensional  Shift (IDS) | Odor | Digging medium | O3 | M3,M4 | O4 | M3,M4 |
| Extra dimensional shift (EDS) | Digging medium | Odor | M5 | O5,O6 | M6 | O5,O6 |

Table1: Types of Stimuli and varied combinations used in the different attentional tasks

*Data analysis*: The number of trials required for each mouse to reach criterion in every task were recorded and then analysed using one-way ANOVA. All tasks were done by an investigator blind to the genotype of the animals.

**Y-maze**

The Y-maze was used to test both spontaneous alternation^5^ and forced delay memory tasks^6^.The apparatus consisted of 3 arms made of opaque black plastic arranged at 120° angle in the shape of a “Y”. The walls of the arms were identical to avoid any cues, additionally the height of the arms were 8cm, which is high enough for the mouse to be able to see distal spatial landmarks.

*Spontaneous alternation*

Mice were placed in the centre of the maze and allowed to freely explore all three arms for 10 min. Since this was the first time the mice encountered the Y-maze, there could be a certain degree of anxiety, so the trial period was assessed as two 5min periods, where the first 5min were considered as habituation period and only the number of entries during the second half of the session were recorded. The spontaneous alternation was calculated as the number of entries in all three arms divided by the total number of entries multiplied by 100. The analysis was automated by the video tracking software, SMART (Bioseb, France). The sessions were recorded for further offline analysis.

*Delayed alternation*

This task involved two phases, a training phase and a test phase. During the training phase, one of the arms was closed and the mouse was placed in one of the two remaining arms which was labelled as the START arm and then allowed to explore for 10min. The START arms and the shut off arms were changed between animals. Then after a delay of 1hr, the mice were reintroduced in the Y maze, this time with all three arms open for exploration. The test phase lasted for 5min. The number of entries and the alternation was calculated as mentioned above.

**Spatial and temporal recognition memory tasks^7^**

Spatial and temporal recognition memory tasks were carried out in an opaque plexiglass box (50cm X 50cm). The floor was covered with sawdust and a video camera was setup over the box, to record the sessions for later analysis. The objects used were of varied shapes, sizes and materials and were stuck to the floor of the box during sessions to avoid displacement. During each interval, all objects and the box were thoroughly cleaned to remove possible odorous cues. In all tasks, the positions of the objects were counterbalanced between mice. Before beginning each of the following tasks, the animals were habituated 15 min to the box for 2 days. Exploration was recorded when the nose of the mouse was within 1cm of an object. Standing over or climbing objects was not considered as exploration.

*Object in place task*

Mice were exposed to a training phase and a test phase separated by a 5min delay. During the training phase, mice were placed in the centre of the box with 4 different objects located 10cm from the walls and allowed to explore freely for 10min. During the test phase, two of these objects were swapped in their position and the mouse was reintroduced for 3min. The discrimination ratio was calculated as the difference in time spent on the objects that changed positions as compared to the time spent on the objects in the same position relative to the total amount of time spent in exploration.

*Temporal order task*

This task is a PFC dependent recency discrimination task, where the animals ideally explore objects encountered at an earlier time point as opposed to a more recently encountered object. Mice were exposed to 2 training phases of 10min each and to a test phase of 3min. Each phase was recorded after a delay of 5min. During the training session, mice were exposed to two copies of the same object. During the second training session, mice were similarly exposed to another set of two copies of a different object. Following a 1hr delay, the test phase included an object from each of the training sessions. The discrimination ratio was calculated as the difference in time spent on an object of training session 1 compared to the object from training session 2 relative to the total amount of time spent in exploration.

*Object Location task*

This task is a hippocampus based spatial memory discrimination task, including a training phase and a test phase after a delay of 5min. During the acquisition phase, the subject mouse was exposed to two objects placed in a straight row, 10cm from the opposite walls of the box for 10min. The amount of time spent on each object was noted by the experimenter. The animal was returned back to the home cage during the delay period, following which, they were reintroduced in the box for 3min. During this test phase, one of the objects was in the original position, while the other object was moved such that the two objects were now in diagonally opposite. The discrimination ratio (DR) was calculated as the difference in the time spent by the mouse exploring the object that changed location to the object in original location relative to the total amount of time spent in exploration.

**Prepulse Inhibition** **(PPI)**

Mice were tested using PPI protocol and apparatus described in Le Pen et al., (2003)^8^. The apparatus comprised 4 startle chambers (S-R LAB, San Diego), each containing a transparent Plexiglas tube (diameter 3.8cm, length 12.7cm) mounted on a frame. A piezoelectric accelerometer was mounted below the frame to detect motion within the tube. Bursts of acoustic noise were presented via a speaker mounted 24cm above the tube while a background noise of 68dB was maintained throughout the session. Startle amplitudes were defined as the average of 100, 1ms stabilimeter readings collected from stimulus onset. Each mouse was put into the PPI chamber for a 5min habituation with 68dB background noise. Following this period, 10 startle pulses (120dB, 40ms duration) were presented with an average inter trial interval of 15sec and over the next 20min, 10 random sequence of no stimulus (background noise, 68dB), prepulse alone (72, 76, 80, or 84 dB, 20ms duration), startle pulses alone, or prepulse followed by startle pulses after a delay of 80ms were presented. The percentage of PPI induced by each prepulse intensity was calculated as 100[(SP−SPP)/SP], with SP being the average startle amplitude after the startle pulses alone and SPP being the average startle response after the combination of a certain prepulse intensity and the startle pulse.

**Social interaction task**

All animals were isolated 3 weeks prior to the task. The apparatus consisted of a transparent Plexiglas box (50cm X 30cm X 30cm) with sawdust on the floor. A video camera was attached above the box to record the interaction. On the day of the task, each test mouse was first habituated to the apparatus for 30min. These mice were called as the “Isolated Host” mice (IH mice). After the habituation, another mouse called as the “visitor; (V)” was introduced in the box. The V mouse belonged to a group-housed cage and was age, weight and gender matched to the IH mouse. Different V mice were used for all the IH mice tested. The interaction between the IH and V was recorded for 8min and the first 4 min were analysed offline for duration and number of social contacts using the MiceProfiler software^9^. The analyses were divided into contact events, relative positions between mice, dynamic events initiated by the IH or the V and stop events. Additional offline analyses were also made to score dominance based on the number of paw control (i.e. the number of times IH placed its forepaw on the back or head of the V mouse) and aggressive display (based on number of tail rattling and bites) by the IH mouse.

**Modified open Field task^10^**

Animals were assessed in overall locomotion as well as anxiety to sudden light in this modified open field task. The apparatus consisted of a Plexiglas box (45×30×45cm; Med Associate). One of the wall of this box was transparent, where a lamp was placed which when switched on, casted an illumination gradient in the box. All other walls as well as the floor were made of opaque Plexiglas. In the beginning of the task, mice were placed in the centre of the box in the dark for 8min followed by the presentation of bright light for the next 4min. Locomotor and rearing activity was estimated based on infrared beam breaks and quantification of faecal drops. The change in locomotor activity in response to light was calculated as the difference in distance travelled between minute 12 and 13 of the test. Additionally, the time spent in the zone farthest from the lamp (11.2cm X 45cm) was considered as dark preference.

**References**

1. Didriksen M, Fejgin K, Nilsson SR, Birknow MR, Grayton HM, Larsen PH*, et al*. Persistent gating deficit and increased sensitivity to NMDA receptor antagonism after puberty in a new mouse model of the human 22q11.2 microdeletion syndrome: a study in male mice. *Journal of psychiatry & neuroscience : JPN* 2017; **42**(1)**:** 48-58.

2. Pittaras E, Cressant A, Serreau P, Bruijel J, Dellu-Hagedorn F, Callebert J*, et al*. Mice Gamble for Food: Individual Differences in Risky Choices and Prefrontal Cortex Serotonin. *Journal of Addiction Research & Therapy* 2013; **S4**( 011).

3. Bissonette GB, Martins GJ, Franz TM, Harper ES, Schoenbaum G, Powell EM. Double dissociation of the effects of medial and orbital prefrontal cortical lesions on attentional and affective shifts in mice. *The Journal of neuroscience : the official journal of the Society for Neuroscience* 2008; **28**(44)**:** 11124-11130.

4. Papaleo F, Crawley JN, Song J, Lipska BK, Pickel J, Weinberger DR*, et al*. Genetic dissection of the role of catechol-O-methyltransferase in cognition and stress reactivity in mice. *The Journal of neuroscience : the official journal of the Society for Neuroscience* 2008; **28**(35)**:** 8709-8723.

5. Hughes RN. The value of spontaneous alternation behavior (SAB) as a test of retention in pharmacological investigations of memory. *Neuroscience and biobehavioral reviews* 2004; **28**(5)**:** 497-505.

6. Yang ST, Shi Y, Wang Q, Peng JY, Li BM. Neuronal representation of working memory in the medial prefrontal cortex of rats. *Molecular brain* 2014; **7:** 61.

7. Manago F, Mereu M, Mastwal S, Mastrogiacomo R, Scheggia D, Emanuele M*, et al*. Genetic Disruption of Arc/Arg3.1 in Mice Causes Alterations in Dopamine and Neurobehavioral Phenotypes Related to Schizophrenia. *Cell Rep* 2016; **16**(8)**:** 2116-2128.

8. Le Pen G, Kew J, Alberati D, Borroni E, Heitz MP, Moreau JL. Prepulse inhibition deficits of the startle reflex in neonatal ventral hippocampal-lesioned rats: reversal by glycine and a glycine transporter inhibitor. *Biological psychiatry* 2003; **54**(11)**:** 1162-1170.

9. de Chaumont F, Coura RD, Serreau P, Cressant A, Chabout J, Granon S*, et al*. Computerized video analysis of social interactions in mice. *Nat Methods* 2012; **9**(4)**:** 410-417.

10. Godsil BP, Stefanacci L, Fanselow MS. Bright light suppresses hyperactivity induced by excitotoxic dorsal hippocampus lesions in the rat. *Behavioral neuroscience* 2005; **119**(5)**:** 1339-1352.


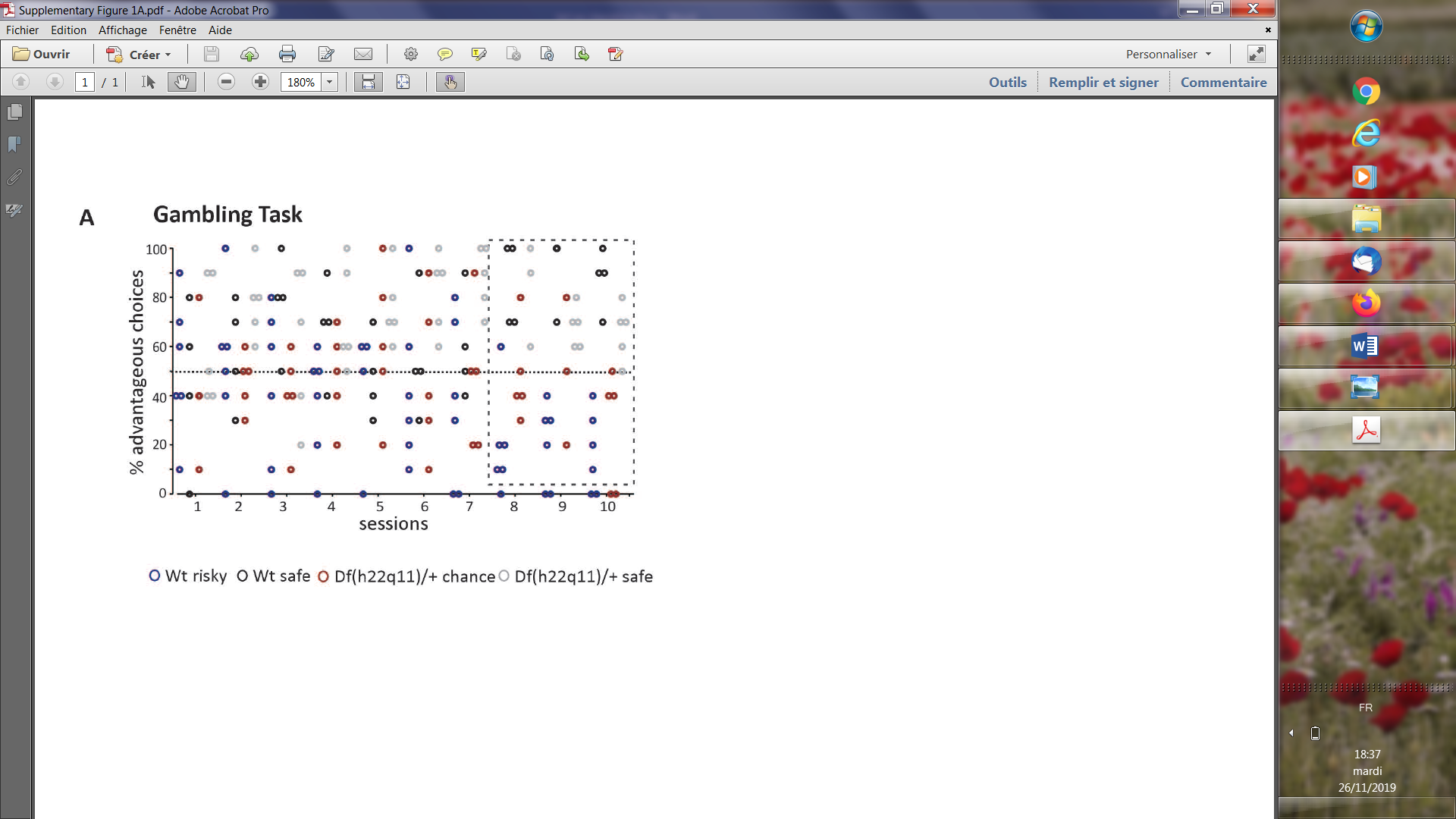


**Supplementary Fig 1A: Df(h22q11)/+ mice show difference in probabilistic learning in the PFC-dependent gambling task.** Open circles in the graph represent the individual performance of different subgroups of Wt and Df(h22q11)/+ mice over 10 days. The animals were subdivided into risky or safe categories based on their choice of advantageous or disadvantageous arms over chance level (denoted by dashed line). Statistical analysis was only performed on the average performance of the last three days (denoted by the dashed box) and not on the individual choices.
