# Supplementary figures and images for: Cognition- and circuit-based dysfunction in a mouse model of 22q11.2 microdeletion syndrome: effects of stress
Source: Transl Psychiatry. 2020 Jan 28;10:41. doi: 10.1038/s41398-020-0687-z (PMC7026063; doi:10.1038/s41398-020-0687-z)

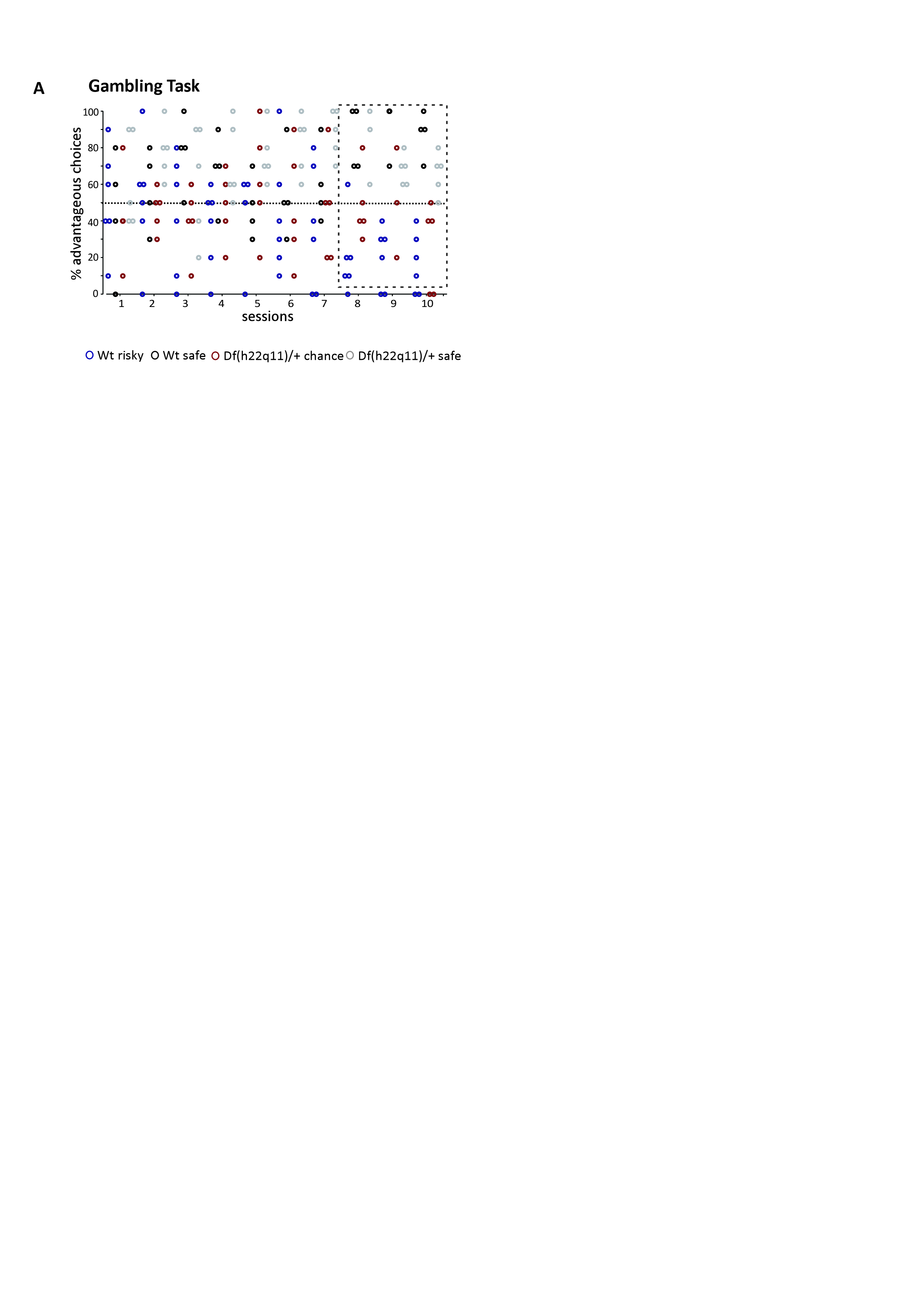

Supplement: Supplementary file 2 — Supplementary Fig 1A [file 41398_2020_687_MOESM2_ESM.tif]
